# Optimization of ribosome utilization in *Saccharomyces cerevisiae*

**DOI:** 10.1093/pnasnexus/pgad074

**Published:** 2023-03-09

**Authors:** Mahima, Ajeet K Sharma

**Affiliations:** Department of Physics, Indian Institute of Technology Jammu, Jammu 181221, India; Department of Physics, Indian Institute of Technology Jammu, Jammu 181221, India; Department of Biosciences and Bioengineering, Indian Institute of Technology Jammu, Jammu 181221, India

## Abstract

Resource optimization in protein synthesis is often looked at from the perspective of translation efficiency—the rate at which proteins are synthesized from a single transcript. The higher the rate of protein synthesis, the more efficiently a transcript is translated. However, the production of a ribosome consumes significantly more cellular resources than an mRNA molecule. Therefore, there should be a stronger selection pressure for optimizing ribosome usage than translation efficiency. This paper reports strong evidence of such optimization which becomes more prominent in highly expressed transcripts that consume a significant amount of cellular resources. The ribosome usage is optimized by the biases in codon usage and translation initiation rates. This optimization significantly reduces the ribosome requirement in *Saccharomyces cerevisiae*. We also find that a low ribosome density on mRNA transcripts helps optimize ribosome utilization. Therefore, protein synthesis occurs in a low ribosome density regime where translation–initiation is the rate-limiting step. Our results suggest that optimizing ribosome usage is one of the major forces shaping evolutionary selection pressure, and thus provide a new perspective to resource optimization in protein synthesis.

**Significance**

Translation efficiency has been traditionally used as a measure of how efficiently proteins are synthesized from a specific transcript. It is defined as the rate of protein synthesis from a single copy of a mRNA transcript. However, since the production of a ribosome consumes much more cellular resources than an mRNA molecule, a better resource optimization can be achieved if ribosome utilization is optimized. We report clear evidence for such an optimization in protein synthesis. We also show that the ribosome utilization is better optimized than the translation efficiency. We find that the biases in codon usage and translation initiation rates optimize ribosome utilization in protein synthesis. Thus, our results provide a new perspective to resource optimization in protein synthesis.

## Introduction

Protein synthesis is a resource-intensive process. It consumes a significant amount of resources and can use up to 50% of the total energy of a cell (1–4). Therefore, protein synthesis is heavily optimized for the efficient use of translation machinery (5, 6). Such optimization is often quantified by translation efficiency (TE)—the rate at which proteins are produced from a single copy of the transcript (7, 1, 8–10). The underlying assumption behind defining TE in this manner is that gene translation becomes more efficient if more proteins are produced from a single transcript. Indeed, a large number of studies have shown that evolutionary selection pressure has optimized TE by introducing several conserved and non-random features in the genetic material (7, 11, 8, 12, 13, 1).

TE quantifies the efficient use of a transcript in protein synthesis (1, 14, 8). The higher the rate of protein synthesis, the more efficiently a transcript is translated. However, the production of a ribosome consumes significantly more energy and nutrients than an mRNA molecule (15, 16). It is also worth mentioning that a considerable portion of the proteome and RNA molecules are required to produce the overall ribosome machinery of a cell (17–19). Therefore, we argue that there should be a stronger selection pressure for optimizing ribosome usages than TE. We can understand this by the following hypothetical example. Consider two mRNA transcripts of the same length. The first mRNA produces three copies of a protein per minute, and the second one produces four copies. Also, assume that the average number of ribosomes on the first and second mRNA transcripts are 3 and 12, respectively. In this example, the second mRNA transcript has higher TE than the first one. This means that translation of the second transcript occurs more efficiently than the first one, while ignoring the fact that it requires four times more resources to produce 33% more proteins. Thus, protein synthesis from the first transcript (i.e. the mRNA with lower TE) is energetically less expensive, because producing a ribosome, that contains tens of RNAs and protein molecules (20), consumes much more energy and other resources than producing an mRNA molecule. This hypothetical example suggests that optimizing ribosome utilization by a transcript could better optimize the overall resource consumption in protein synthesis than TE. In addition to that, at a given time around 85% of the total *Saccharomyces cerevisiae* ribosomes are actively engaged in protein synthesis under normal growth conditions (21–24). Therefore, the optimization of ribosome utilization by mRNA transcripts must have a significant impact on the overall ribosome requirement of a cell. For these reasons, further investigations are required to understand how an mRNA transcript optimizes ribosome utilization and its broader implications on overall protein synthesis.

In this paper, using the transcriptome-wide simulations of protein synthesis in *S. cerevisiae*, we show the evidence of the optimization of ribosome usage in protein synthesis. We find that ribosome utilization is better optimized than TE, especially in transcripts that consume more resources in protein synthesis. The ribosome utilization is optimized by codon usage bias and non-random distribution of translation–initiation rates among different transcripts. This optimization significantly decreases the overall ribosome requirement in *S. cerevisiae*. We also find that the protein production from *S. cerevisiae* transcripts is much lower than their peak production capacity. It is because the decreased protein production allows efficient optimization of ribosome usages. These results demonstrate that the optimization of ribosome usage is a significant contributor to evolutionary selection pressure shaping the overall translational landscape of an organism.

## Materials and methods

### Modeling protein synthesis

We simulate protein synthesis using the totally asymmetric simple exclusion process (TASEP) model. This model and its various extensions have been widely used over the past 50 years to simulate in vivo protein synthesis (25–33). TASEP model represents mRNA as a one-dimensional lattice where each site in the lattice can be thought as a single codon (34–36). Thus, the total number of sites in the lattice is equal to the number of codons in the coding sequence of the mRNA molecule. A ribosome in this model covers 10 successive codon positions (37, 38, 35, 39). Therefore, we use ribosome A-site to denote its position on an mRNA transcript.

**Fig. 1.**
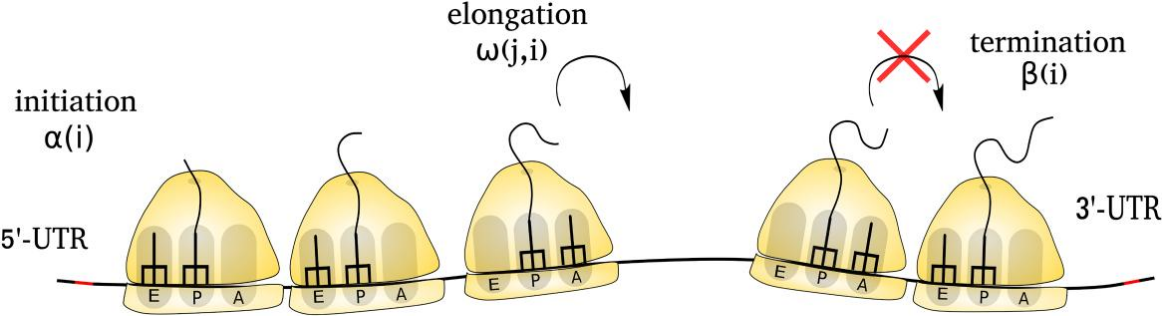
A pictorial depiction of the process of protein synthesis. Protein synthesis occurs in three sequential steps: (1) initiation, (2) elongation, and (3) termination. Initiation in transcript *i* occurs when a ribosome arrives at the start codon with rate α(i). During elongation, a ribosome moves towards the stop codon by taking a series of stochastic steps. In each of such steps, it jumps to the next codon and elongates the nascent protein by one amino acid subunit. A ribosome moves from codon position *j* to j+1 with a rate ω(j,i). The termination of protein synthesis occurs with rate β(i) when the ribosome reaches the stop codon.

In our model, protein synthesis initiates with rate α(i) when both ribosome subunits assemble at the start codon (Fig. 1). This happens only if the first six codons of the mRNA transcript are not occupied by any other ribosome (40). Then, during the elongation step, the ribosome starts moving unidirectionally in a stochastic manner. It hops from codon position *j* to j+1 with rate ω(j,i). A ribosome incorporates an amino acid into the growing nascent-chain as it moves to the next codon position. During elongation, no two ribosomes can occupy the same site (codon). Therefore, a ribosome cannot overtake another ribosome while moving on the mRNA transcript. Protein synthesis in this model is terminated with rate β(i) when a ribosome encounters the stop codon. We simulated this model using the Gillespie’s algorithm described in Refs. (41, 42).

### Estimation of the requirement of additional ribosomes if non-randomness in the codon usage and translation initiation rates are eliminated

We have quantified the requirement of additional ribosomes for the same amount of protein production if the biases in codon usage and translation–initiation rates are eliminated. To this end, we have used two different sets of transcripts. The first set consists of the wild-type *S. cerevisiae* transcripts. The second set is created by mutating each codon of a transcript with a randomly selected synonymous codon. For the wild-type transcripts, we have used the translation initiation rates reported in Refs. (43, 42), whereas for the other set, we have randomly redistributed those initiation rates among all *S. cerevisiae* transcripts. Thus, we have eliminated the biases in codon usage and translation initiation rates in the second set of transcripts.

In our calculations, we denote the TE for *i*th wild-type and mutated transcripts as ${\mathrm{TE}}_{i}$ and ${\mathrm{TE}}_{i}^{\prime }$, respectively. Similarly, the average number of ribosomes on the wild-type and mutated transcripts are denoted by $N_{i}$ and $N_{i}^{\prime }$, respectively. In the absence of any biases in codon usage and initiation rates, $N_{i}^{\prime }$ ribosomes synthesize ${\mathrm{TE}}_{i}^{\prime }$ proteins per second on a single copy of the *i*th mutated transcript. This means that a single protein per second is produced by $N_{i}^{\prime }/{\mathrm{TE}}_{i}^{\prime }$ ribosomes, and thus the production of ${\mathrm{TE}}_{i}$ proteins per second would require $(N_{i}^{\prime }/{\mathrm{TE}}_{i}^{\prime }){\mathrm{TE}}_{i}$ ribosomes. Hence, the percentage of additional ribosomes required in protein synthesis for *i*th transcript is


(1) $$\gamma_{i}=\frac{\left((N_{i}^{\prime }/{\mathrm{TE}}_{i}^{\prime }){\mathrm{TE}}_{i}-N_{i}\right)}{N_{i}}\times 100.$$

Further simplification of Eq. 1 results in


(2) $$\gamma_{i}=\left(\frac{{\mathrm{RUE}}_{i}}{{\mathrm{RUE}}_{i}^{\prime }}-1\right)\times 100.$$

In Eq. 2, ${\mathrm{RUE}}_{i}$ and ${\mathrm{RUE}}_{i}^{\prime }$ are the ribosome utilization efficiencies for the wild-type and mutated transcripts, respectively. RUE is defined as follows:


(3) $$\mathrm{RUE}=\frac{\mathrm{TE}}{N}L,$$

*L* in Eq. 3 is the length of the coding region. See the result section for the rationale behind defining RUE in this manner.

We also calculate the total number of additional ribosomes required to achieve the same amount of protein synthesis with a non-biased transcriptome. We calculate it by summing over the additional number of ribosomes required for each copy of all transcripts. Thus, the overall percentage of additional ribosomes required in the absence of any biases in codon usage and initiation rate is


(4) $$\Gamma =\sum_{i}\gamma_{i}n_{i}=\sum_{i}\left(\frac{{\mathrm{RUE}}_{i}}{{\mathrm{RUE}}_{i}^{\prime }}-1\right)n_{i}\times 100.$$

In Eq. 4, $n_{i}$ is the mRNA copies belonging to the *i*th transcript and is calculated using the RNA-Seq read per kilobase per million (RPKM) values reported in Ref. (44).


(5) $$n_{i}=\frac{{\mathrm{RPKM}}_{i}}{\sum_{i}{\mathrm{RPKM}}_{i}}\left(\sum_{i}n_{i}\right),$$

$\sum_{i}n_{i}$ in Eq. 5 is the total number of mRNA molecules in *S. cerevisiae*. Its typical value is 40,000 (45).

### Measurement of the transcript-specific TE and RUE

To calculate TE, we simulate protein synthesis on a transcript and measure the time it takes to complete the production of 10,000 proteins. Then, dividing 10,000 with this synthesis time gives the rate of protein synthesis, also known as the TE. To compute RUE, we require already calculated TE, average number of ribosomes on a transcript (*N*), and the number of codons in the coding sequence (*L*) of the mRNA (Eq. 6). To calculate *N*, first we wait for the translation system to achieve the steady state. Then, we take 10,000 snapshots of the translation system. Each such snapshot gives the location of ribosomes translating that specific transcript which we use to calculate average ribosome density on a transcript. Then, we compute RUE by inserting *N*, TE, and *L* in Eq. 6.

## Results

### RUE is defined as the rate of amino acid addition by a single ribosome

We quantify the efficiency of ribosome utilization by introducing a parameter—RUE.


(6) $$\mathrm{RUE}=\frac{\mathrm{TE}}{N}L.$$

In Eq. 6, TE is the translation efficiency, *N* is the average number of ribosomes on the transcript, and *L* is the number of codons in the coding sequence. The rationale behind defining RUE in this manner is the following. Normalizing TE with *N* gives the contribution of a single ribosome in the overall rate of protein production from an mRNA transcript. However, the length of the coding sequences varies significantly among different transcripts. This makes TE/N length-dependent as *N* will be larger in longer transcripts. Therefore, we offset this bias by multiplying TE/N with the length of the coding sequence (Eq. 6). This makes RUE the rate of amino acid addition by a ribosome. The main difference between TE and RUE is that the TE measures the efficiency of protein synthesis from a single transcript (1, 5, 8–10), whereas RUE quantifies how efficiently a ribosome translates a specific transcript.

**Fig. 2.**
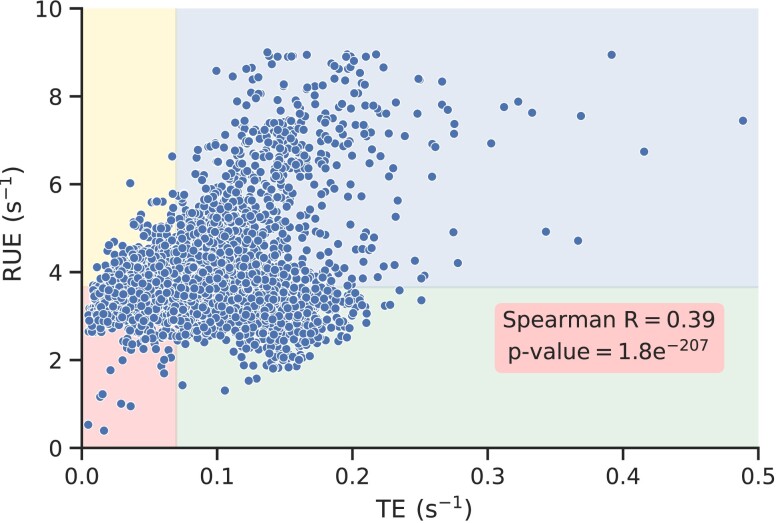
A transcriptome-wide comparison of TE with RUE. RUE of S. cerevisiae transcripts are plotted against the TE. The figure consists of four subsections which are created by the lines separating them at median TE and RUE.

RUE is a function of TE (Eq. 6) and both of them correlate with each other (Fig. 2). Therefore, there is a possibility that RUE carries no new information. This means, both of these parameters can be used interchangeably. TE and RUE are the same, only if N/L (i.e. average ribosome density (ρ)) remains constant in all *S. cerevisiae* transcripts. However, polysome profiling data reported in Ref. (21) show more than two orders of magnitude variation in the average ribosome density of all *S. cerevisiae* transcripts.

To further rule out this possibility, we apply two different tests. First, we test whether the genes with high TE also have a high RUE. To test this, we simulate protein synthesis on all *S. cerevisiae* transcripts (see Methods section), measure RUE and TE, and plot them against each other (Fig. 2). In this test, if the TE of a gene is greater than the transcriptome-wide median, then the gene is considered as a high TE gene; otherwise the gene has a low TE. Similarly, we label all genes with high or low RUE genes. To clearly visualize this, we divide Fig. 2 in four different regions by the lines separating them at median RUE and TE. We find that 42.2% of the total genes are either in the left top or bottom right regions of Fig. 2. In those genes, RUE (or TE) is high, but TE (or RUE) is low. This means that a low or high TE does not ensure a low or high RUE, respectively. We also compared the TE and RUE of each transcript with the rest of the other transcripts. We find 7,163,405 out of the 17,032,366 gene pairs (i.e. over 42% of the total gene pairs), a gene with a larger (or smaller) TE has a smaller (or larger) RUE.

Second, if RUE is a representative of TE, then both of these parameters should follow the same distribution because there should be no systematic differences between them. To test this, we first normalize TE and RUE with respective mean values and compute their probability distribution (Fig. S1). Then, we test whether the normalized TE and RUE follow the same distribution by applying the Kolmogorov Smirnov test. The test rejects the null hypothesis that normalized TE and RUE are distributed in a similar manner (P- value=1.1321e−277). This shows a systematic difference between TE and RUE. Thus, despite a correlation between TE and RUE, one cannot be used as a proxy for the other.

While simulating protein synthesis for Fig. 2, we used the translation–initiation and codon translation rates reported in Refs. (43, 46), respectively. We also test the robustness of these results by computing RUE and TE from the protein synthesis simulations carried out by using the initiation and codon translation rates reported in Ref. (42), and find similar results (supporting results and Figs. S12 and S13).

### RUE is better optimized in transcripts that require more ribosomes for protein synthesis

**Fig. 3.**
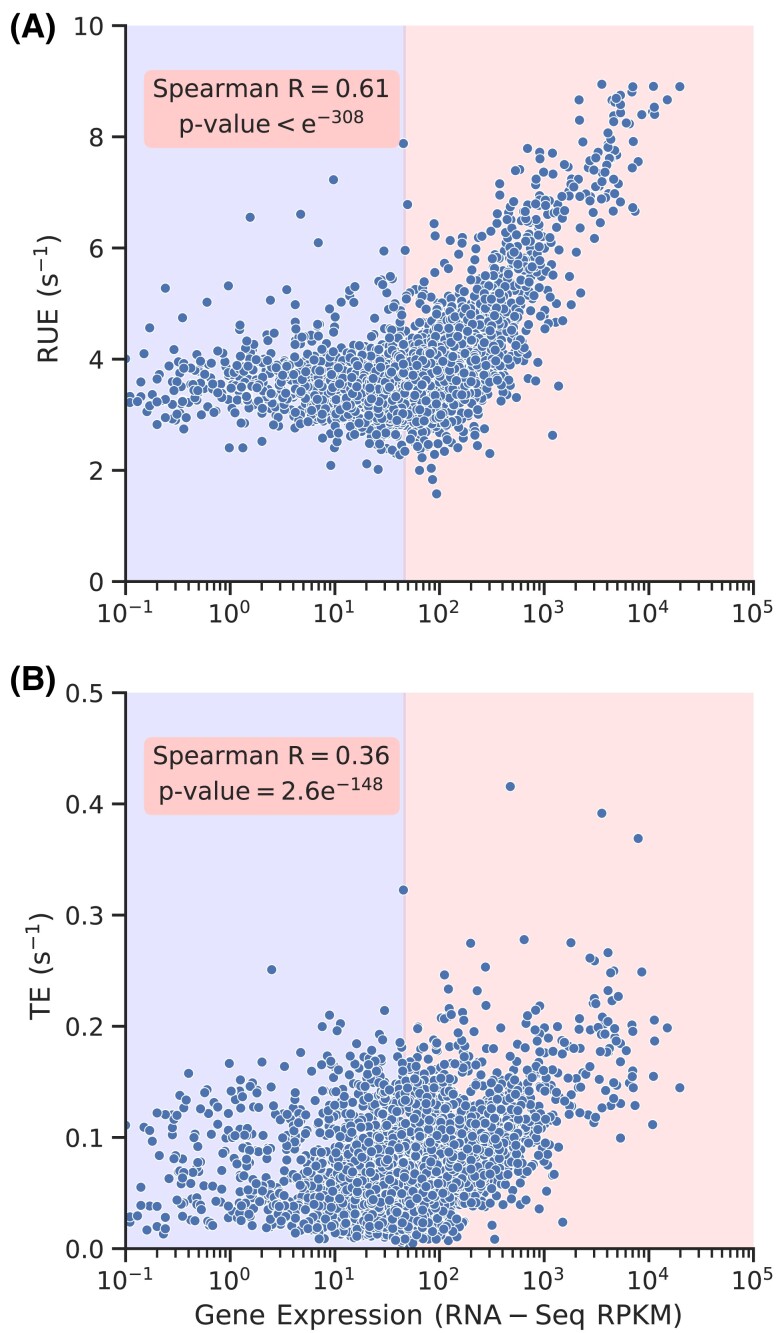
RUE is better optimized than TE. RUE and TE are plotted against mRNA abundance in A and B, respectively. (A) and (B) are divided into two subsections at the median value of mRNA expression levels. Highly expressed genes with RPKM values above median show a better correlation with RUE and TE.

Having established that TE is not a representative of RUE, we hypothesize that RUE is better optimized in highly expressed transcripts. It is because these are the transcripts that consume a major portion of the overall resources used in protein synthesis. We test this by plotting mRNA abundance with RUE and find a positive correlation between them (Fig. 3A). This shows that highly expressed transcripts tend to have stronger optimization of ribosome utilization. Also, the correlation of mRNA abundance with RUE is much stronger than TE (Fig. 3). Similarly, the correlation of protein abundance with RUE is much stronger than TE (Fig. S2). This suggests that RUE is the parameter whose optimization in highly expressed transcripts is more critical than TE. We also notice that there is no correlation between mRNA abundance and RUE in low expressed genes (see the left half portion of Fig. 3A). Spearman correlation in the data shown in the light-blue portion is 0.038 with a *P*-value of 0.052. It is because the requirement for the optimization of ribosome utilization in those transcripts is very minimal. Thus, selection pressure for optimizing ribosome utilization is very weak.

We found similar results when we computed RUE and TE by simulating protein synthesis using the translation–initiation and codon translation rates reported in Ref. (42) (Fig. S3). These results demonstrate that highly expressed transcripts have a relatively stronger optimization of ribosome utilization; RUE is better optimized than TE in those transcripts.

### Codon usage bias and non-randomness in initiation rate contribute in optimizing RUE

Having established that RUE is better optimized in a transcript that consumes more resources, we ask the question how this parameter is being optimized. Since it is the translation initiation and codon translation rate that control the overall kinetics of protein synthesis (42, 23, 35), we hypothesize that RUE is being regulated by the non-random features of genetic material that can influence those rate parameters. To test this, we plot RUE as a function of translation–initiation rate and codon adaptation index (CAI). (CAI tells the extent of bias for more frequent codons (47, 48).) We find that RUE positively correlates with translation–initiation and CAI, suggesting their positive contribution to the optimization of RUE (Fig. S4).

**Fig. 4.**
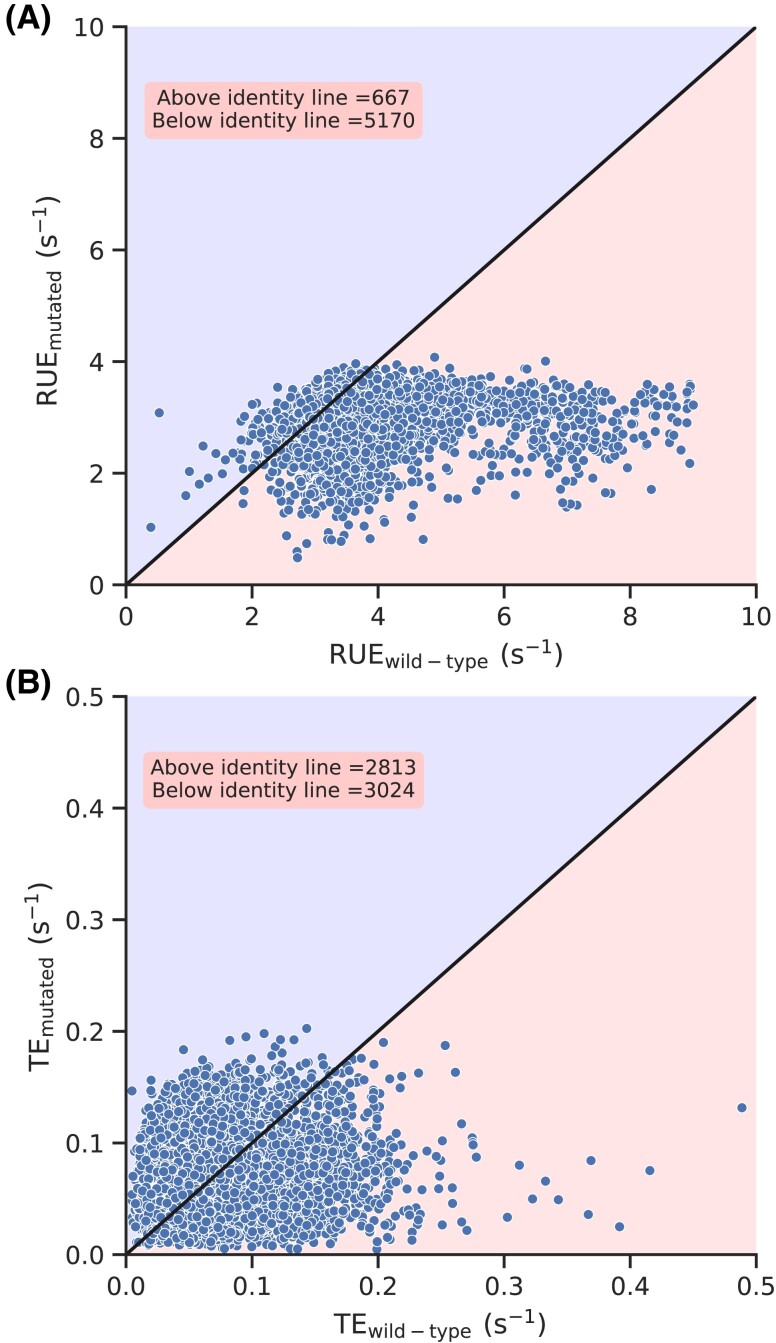
Effect of randomizing codon usage bias and initiation rates. RUE and TE of the wild-type transcripts are plotted against the RUE and TE of mutated transcripts in (A) and (B), respectively.

To further understand their contribution, we created a new set of *S. cerevisiae* transcripts which are different from the wild-type sequences but code for the same proteins. We created these transcripts by mutating each codon of a transcript with a randomly selected synonymous codon. Similarly, we redistributed initiation rate randomly to each of the transcripts (see Methods). Therefore, this new set of transcripts does not have any codon usage bias, nor do they have any biases in translation initiation rate. Then, we simulated protein synthesis on these mutated transcripts and calculated TE and RUE for each of them. We plot RUE of the mutated sequences against their wild-type counterparts and find that the wild-type RUE is greater than the mutated ones in more than 88% of genes (Fig. 4A). We also find that wild-type TE is greater than the TE of the mutated sequence in 51.8% of transcripts (Fig. 4B). This means that eliminating the biases in codon usage and initiation rates decreased the RUE of the majority of the genes. However, as opposed to RUE, TE of almost half of the transcripts is increased. We repeated this analysis by creating 20 different sets of mutated transcripts and found similar results (see Supplementary Table S1). These results show that nature optimizes ribosome utilization through the codon usage bias and non-randomness in translation initiation rates.

**Fig. 5.**
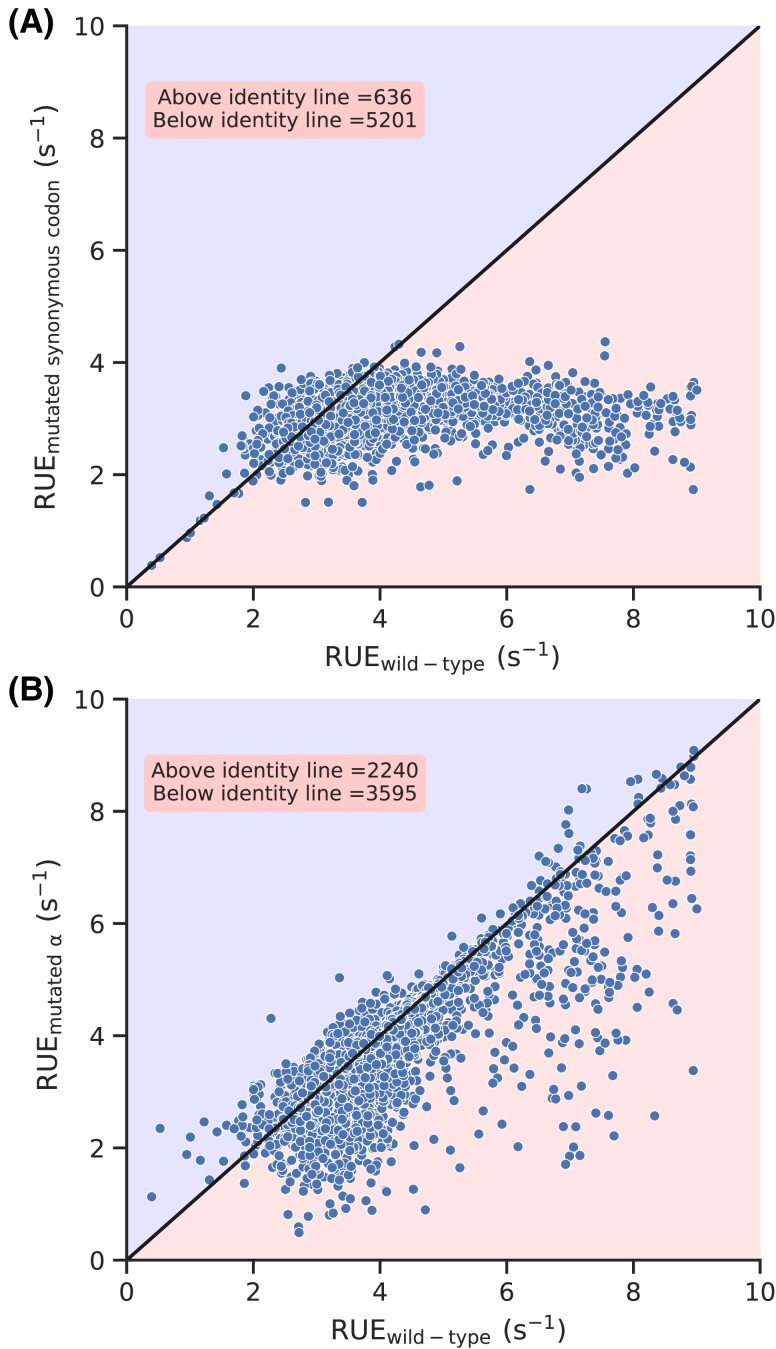
Selection pressure on optimizing codon usage bias is much stronger than translation initiation rate. (A) RUE of the wild-type transcripts are plotted against the RUE of same transcript when all of its codons are mutated with randomly selected synonymous codons. (B) RUE of the wild-type transcripts are plotted against the RUE of same transcript with a randomly assigned initiation rate.

Next, we investigate the individual contributions of codon usage bias and non-random distribution of translation initiation rates in RUE optimization. To this end, first we replaced each codon of all the transcripts with a randomly selected synonymous codon while not changing their wild-type initiation rates. Then, we simulated protein synthesis on those transcripts and measured RUE for each of them. We plot RUE of these mutated transcripts against their wild-type counterparts and find that the random distribution of synonymous codons decreases RUE in more than 89% transcripts (Fig. 5A). Next, we redistributed the measured wild-type initiation rates among different mRNA transcripts without changing their original codon sequence. Then, we simulate protein synthesis on each of these transcripts and compute RUE. We plot the RUE of these mutated transcripts against their wild-type counterparts and find that the random distribution of initiation rate decreases the RUE in 61.5% of transcripts (Fig. 5B). These results show that non-random distribution of codon usage and initiation rates synergistically contribute to the optimization of RUE. However, a greater impact of the elimination of codon usage bias on RUE suggests its more prominent role in optimizing RUE as compared to initiation rate. This observation is also consistent with a stronger correlation of RUE with CAI as compared to the initiation rate (Fig. S4).

We also found similar results when we computed RUE and TE by simulating protein synthesis using the initiation and codon translation rates reported in Ref. (42) (Figs. S5 and S6). Taken together, these results suggest that non-random distribution of codon usage and initiation rates optimizes RUE in *S. cerevisiae* transcripts. We also show that the central utility of codon usage bias is more towards optimizing RUE than TE.

### Codon usage bias and non-random distribution of initiation rates reduce the overall cost of protein synthesis

A higher RUE of a transcript allows producing more proteins using fewer ribosomes. Therefore, there must be some cost benefit for the transcriptome-wide optimization of RUE. We quantify this by calculating the additional number of ribosomes required to produce similar amount of proteins if the optimization of ribosome usage is eliminated.

We find that the percentage of additional ribosomes required for the *i*th gene is


(2) $$\gamma_{i}=\left(\frac{{\mathrm{RUE}}_{i}}{{\mathrm{RUE}}_{i}^{\prime }}-1\right)\times 100.$$

**Fig. 6.**
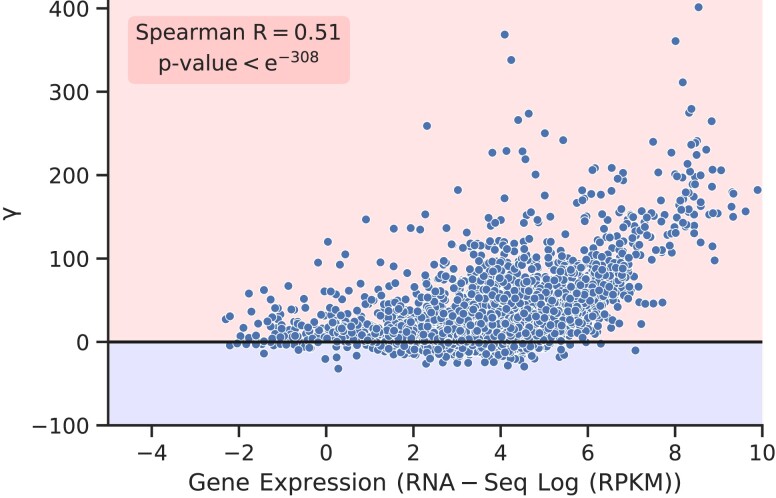
Codon usage bias and non-randomness in initiation rates result in optimizing cost of protein synthesis. The percentage increase in the additional ribosome requirement for a similar amount of protein production is plotted against mRNA abundance. The genes above the γ=0 line require more ribosomes, whereas the genes below this line require less ribosomes, if codon usage bias and non-randomness in the translation elongation rate is eliminated.

${\mathrm{RUE}}_{i}$ and ${\mathrm{RUE}}_{i}^{\prime }$ in Eq. 2 are the RUEs for the *i*th wild-type and mutated sequences, respectively. Note that there is no codon usage bias, and translation initiation rates are randomly distributed in the mutated sequences. Thus, the ribosome usage is not optimized in the mutated sequences. The detailed derivation for $\gamma_{i}$ is provided in the Methods section. We plot $\gamma_{i}$ against mRNA abundance and find that it is positive in more than 90% of transcripts. This means, more ribosomes are required in those transcripts if the biases in codon usage and translation initiation rates are eliminated (Fig. 6). We also find a statistically significant correlation between $\gamma_{i}$ and mRNA abundance (Spearman R=0.51, P- value<e−308). This positive correlation suggests that there is a greater requirement of ribosomes for highly expressed transcripts if non-random kinetic features of the genetic material are eliminated. In fact, the ribosome requirement reaches more than 100% for many highly expressed transcripts. It is because RUE is strongly optimized in highly expressed transcripts (Fig. 3A), and thus any changes in the protein synthesis kinetics can significantly influence the ribosome usage.

We also calculate the total number of additional ribosomes required for a similar amount of protein production if codon usage bias and non-randomness in translation initiation rate are eliminated in all *S. cerevisiae* transcripts. The percentage of additional ribosomes


(4) $$\Gamma =\sum_{i}\left(\frac{{\mathrm{RUE}}_{i}}{{\mathrm{RUE}}_{i}^{\prime }}-1\right)n_{i}\times 100.$$

In Eq. 4, $n_{i}$ is the average mRNA copy number of the *i*th transcript. The detailed mathematical deviation for Γ is provided in the Methods section. We have calculated $n_{i}$ using the RPKM values of RNA-Seq data reported in Ref. (44). The procedure to convert RPKM values to mRNA abundance is also explained in the Methods section. Using Eq. 4, we find that, on average, *S. cerevisiae* requires 52.89±0.31% more ribosomes to produce a similar amount of proteins if biases in codon usage and initiation rates are eliminated. Note that the error bars here are the standard error calculated using Γ from 21 different sets of mutated transcripts. The details of the Γ calculated from each mutated set of transcripts are given in Supplementary Table S1. These results show that the optimization of ribosome usage in *S. cerevisiae* significantly reduces the overall cost of protein synthesis.

### RUE is optimized in a low-density regime

For a transcript with a uniform codon translation rate, ribosome traffic on the mRNA sequence can be categorized into three different phases: low density (LD), high density (HD), and maximal current (MC) (49, 25). In the LD phase, ribosome density on the transcript is low, whereas it is high in the HD phase. MC regime has intermediate ribosome density, but it maximizes the rate of protein synthesis, i.e. TE. Similar three different phases are also seen in non-homogeneous transcripts, where each codon is translated at a different rate (34). If nature had optimized TE, then the protein synthesis would have occurred in the MC regime. However, many experimental and simulation studies have shown that protein synthesis in *S. cerevisiae* occurs in the LD regime (36, 25, 29). Therefore, we wondered whether it is the RUE that is optimized in the LD regime.

**Fig. 7.**
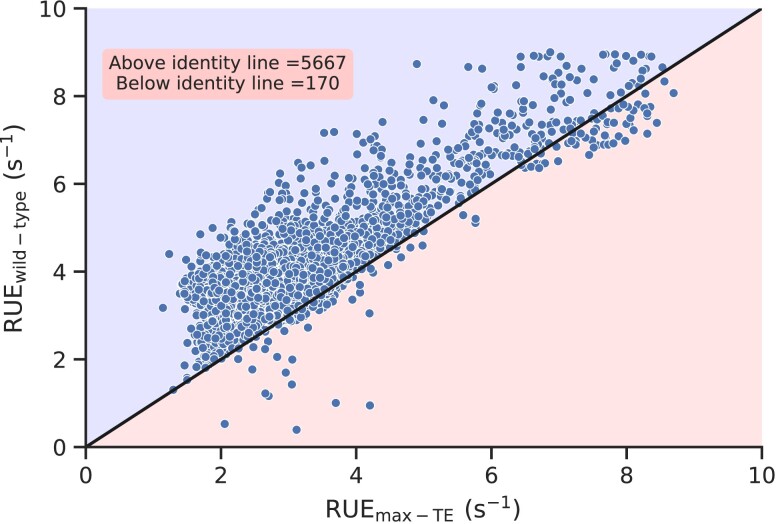
Comparison of the wild-type RUE with the RUE at initiation rate that maximizes TE. Wild-type RUE is plotted against the RUE of a transcript at initiation rate that maximizes TE. Wild-type RUE of 97.08% genes is greater than the RUE at α that maximizes TE.

To understand whether low initiation rates optimize RUE, we vary initiation rate from 0.000 to 4.000 s${}^{-1}$ for each transcript and calculate TE and RUE at each of those initiation rates. We find that increasing initiation rate first increases the TE, and then it saturates as protein synthesis transitions from the LD to MC regime (Fig. S7). However, increasing initiation rate initially increases the RUE, and then a slight dip was observed, showing a maxima in RUE vs. α curve (Fig. S7). This shows that an optimal value of translation–initiation rate maximizes RUE. We then identify the numerical values of initiation rate that maximizes TE and compare the RUE at this initiation rate with the wild-type RUE (Fig. 7). We find that in 97.08% of genes, wild-type RUE is greater than the RUE at initiation rate that maximizes TE. This means, the initiation rates that maximizes TE are different than those that optimizes RUE. Next, we also plot cumulative probability distribution of the ratio of the wild-type initiation rate with the initiation rate that maximizes TE (i.e. the rate at which ribosome traffic transitions from the LD to MC regime). We find that this ratio is 0.75 or lower for more than 95% of the transcript (Fig. S8). Taken together, these results show that wild-type initiation rates that optimize RUE are much lower than the ones that maximizes TE.

**Fig. 8.**
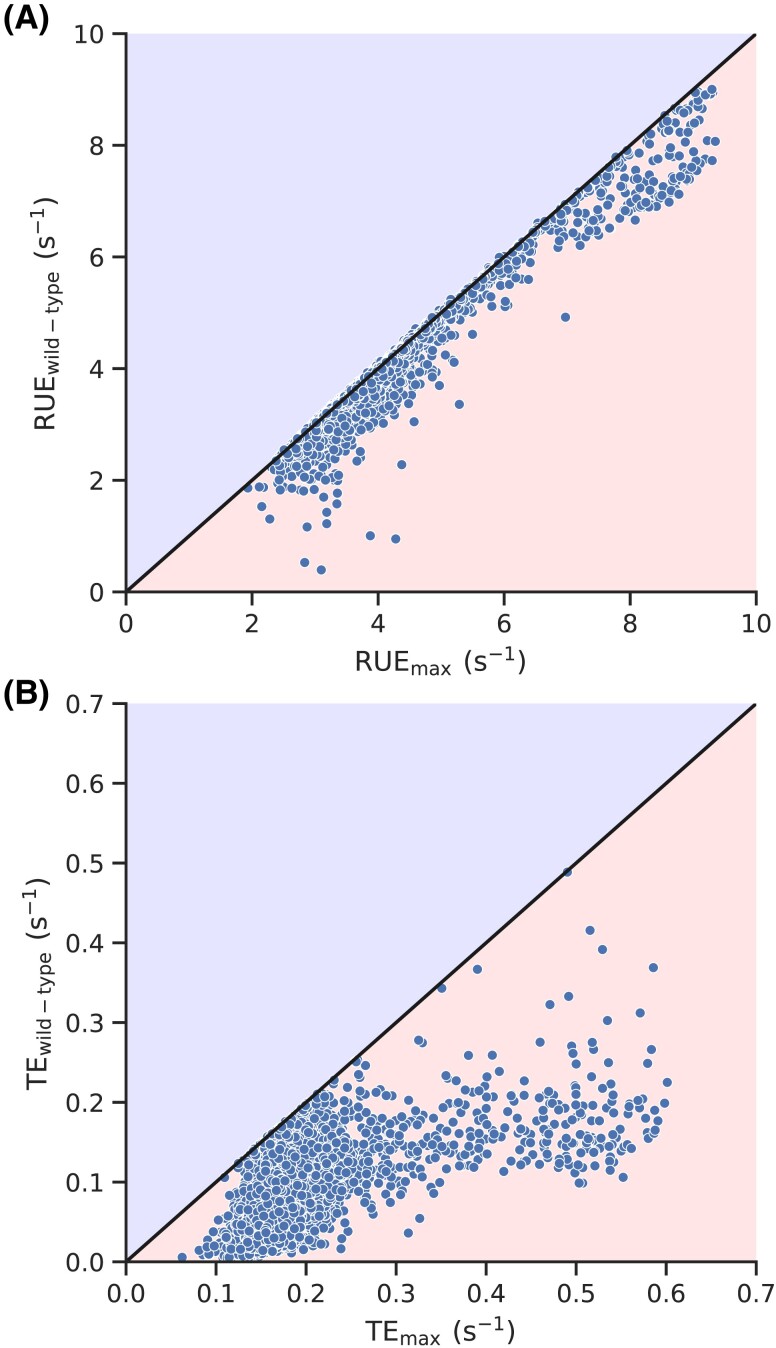
Low initiation rates in *S. cerevisiae* transcript help in optimizing RUE. (A) Wild-type and maximum RUE for all *S. cerevisiae* transcripts are plotted against each other. (B) Wild-type and maximum TE for all *S. cerevisiae* transcripts are plotted against each other.

We next access how close the wild-type RUE and TE are as compared to the maximum RUE and TE. To this end, we vary initiation rate in our simulations and find the maximum RUE and TE for each of the transcripts. Then, we compared the maximum RUE and TE with the wild-type RUE and TE, respectively (Fig. 8). We find that the maximum RUE for a majority of transcripts is very close to the wild-type RUE, which is not the case with TE. We also plot the wild-type initiation rates against the rates that maximize RUE and find a statistically significant correlation between them (Fig. S9).

We find that the selection pressures on initiation rates are much weaker than on codon usage bias (Figs. 5 and S4). This observation suggests that the initiation rate’s overall contribution to RUE optimization is minimal (Fig. 5B). However, we also find that varying initiation rates significantly impact the RUE of a transcript (Fig. S7). Both observations seem to be contradicting each other. Therefore, to further investigate the role of initiation rate in RUE optimization, we create four different sets of transcripts. In each of these sets, we shift the wild-type initiation rate of each transcript by 0.2, 0.4, 0.6, and 0.8 s${}^{-1}$. However, the coding sequence and codon translation rates in each of those four sets are the same as wild-type sequences. The distributions of initiation rate for all of these four sets are plotted in Fig. S10. We then simulate protein synthesis on these four additional sets of transcripts and calculate transcript-specific RUE for each of them. We find that the RUE in 80%, 92.24%, 95.43%, and 96.68% of transcripts decreases when initiation rate is shifted by 0.2, 0.4, 0.6, and 0.8 s${}^{-1}$, respectively (Fig. S11). This observation suggests that RUE optimization requires the initiation rates to be in a specific range where it should be much smaller than the average codon translation rate of a transcript. It is also consistent with the fact that 90% (i.e. between 5th and 95th percentile) of the transcripts have in vivo initiation rates between 0.025 and 0.30 s${}^{-1}$ (43). This range is very narrow as compared to the average codon translation rate in *S. cerevisiae*, i.e. 5 AA/s (42). Thus, randomizing the initiation rate will not have a severe impact on ribosome utilization as long as they are much slower than the average codon translation rate (Fig. 5B).

### Biological implications of RUE optimization

**Fig. 9.**
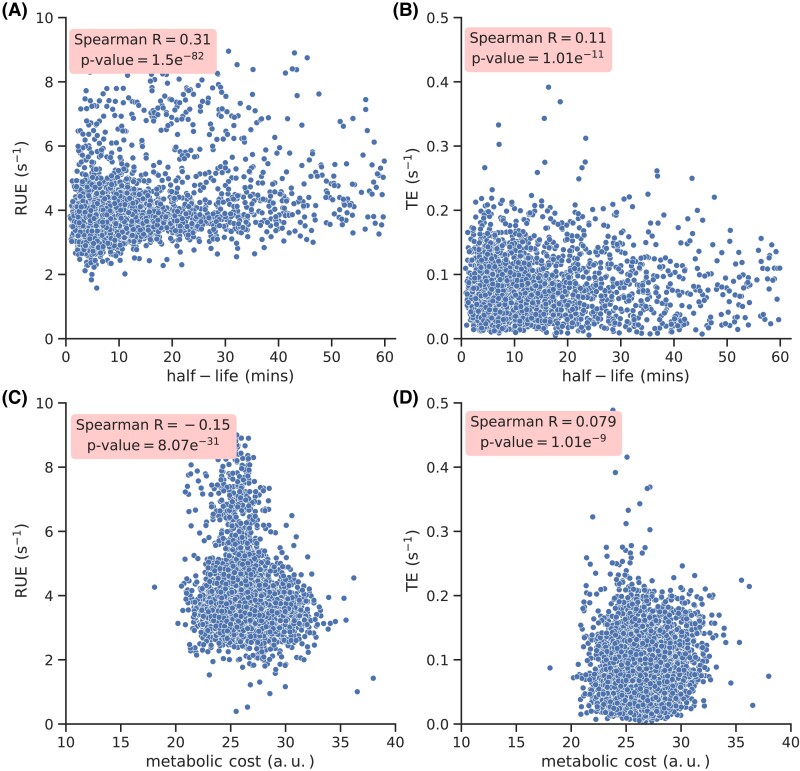
mRNA half-life and production cost of all amino acids in a protein correlate with RUE. mRNA half-life reported in Ref. (50) are plotted against RUE and TE in (A) and (B), respectively. Production cost of all amino acids (51) in a protein are plotted against the RUE and TE in (C) and (D), respectively.

We show that ribosome utilization is better optimized than TE (Fig. 3). However, to achieve a greater optimization of resources, it is possible that other translation-related processes may have also tuned themselves to work synergistically with ribosome utilization. To test this, first we understand whether mRNA half-lives in *S. cerevisiae* are tuned according to the strength of RUE optimization. To this end, we plot mRNA half-life of each transcript against the RUE and TE. We find a stronger positive correlation of RUE with mRNA half-life as compared to TE (Fig. 9A and B). This shows that the degradation machinery tends to degrade inefficient transcripts at a faster rate than others.

Different amino acids have varying production cost, which has been quantified in Ref. (51). This dataset allows us to compute the production cost of all amino acids of a protein. Using this, we plot the amino acid production cost of a protein against the wild-type RUE and TE (Fig. 9C and D). We find that the amino acid production cost of a protein correlates negatively with RUE; however, the correlation is positive with TE. This observation suggests that transcripts that are better optimized for ribosome utilization tend to code for proteins with a lesser metabolic cost.

**Fig. 10.**
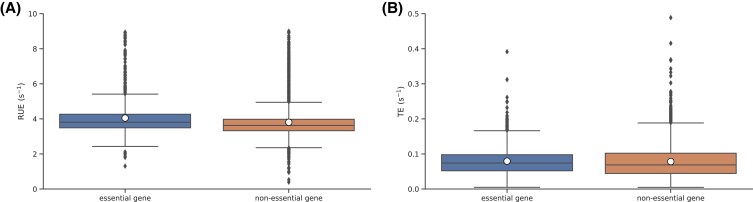
RUE and TE are better optimized in genes that code for essential proteins. Box-plots for RUE and TE of essential and non-essential genes are shown in (A) and (B), respectively. Average RUE in essential genes is 5.1% higher than others whereas, average TE is only 1.4% higher. In this figure, we use the list of essential and non-essential genes reported in Ref. (52).

We also tested whether RUE is better optimized in transcripts that code for essential proteins. These are the proteins that are critical for the survival of an organism. Therefore, we expect such genes to have better optimization of ribosome usage. Indeed, we found that the average RUE was 5.1% higher in essential genes than the others (Fig. 10A; Wilcoxon rank-sum test, P- value=1.1827e−22). However, the difference between the average TE of the essential and non-essential genes was only 1.4% (Fig. 10B; Wilcoxon rank-sum test, P- value=0.0052).

Taken together, these results show that multiple strategies work in tandem for optimizing the overall cost of protein synthesis. We found similar results (Fig. S14) after computing RUE and TE from the protein synthesis simulations carried out using the other dataset of initiation and codon translation rates reported in Ref. (42).

## Discussion

Protein synthesis is central to the survival and proliferation of a cell. It is a resource-intensive process that consumes up to 50% of the total energy of a cell (3, 2, 53). Therefore, evolutionary selection pressure must have optimized resource utilization in protein production (54). However, there was no clarity on how exactly energy and nutrients consumption are optimized for protein synthesis. In this paper, we address this question and show that natural selection has optimized ribosome utilization as a significant amount of energy resources goes into the production of proteins and RNA molecules that make a ribosome (55). We study the optimization of ribosome utilization by introducing a parameter—RUE that measures how efficiently a ribosome synthesizes proteins from a specific transcript. Using this parameter, we show the strong evidence of the optimization of ribosome usage in *S. cerevisiae*. The ribosome utilization is more efficiently optimized in highly expressed transcripts because any inefficiency in those transcripts would drain more resources than the other transcripts (56). We also show that the biases in codon usage and translation–initiation rates optimize the ribosome utilization which significantly reduces their requirement in *S. cerevisiae*.

The RUE we have defined may have some overlap with the TE, but both of these quantities are significantly different from each other (Fig. 2). This difference is also manifested when we look at how and why RUE is optimized. For example, randomizing initiation and codon translation rates eliminates the optimization of RUE and significantly increases the cost of protein synthesis (Figs. 4A and 6). However, such randomization has no significant impact on TE optimization (Fig. 4B). Similarly, we see a very strong optimization of RUE in highly expressed transcripts; RUE in lowly expressed transcripts is not optimized. However, we do not find such a clear distinction between TE optimization in highly and lowly expressed genes (Fig. 3). These results show that RUE and TE have different characteristics, and *S. cerevisiae* prefers to optimize ribosome utilization over protein production. Moreover, codon usage bias has been traditionally believed to optimize TE (6). However, we find that eliminating codon usage bias has minimal impact on TE whereas it significantly decreases RUE, showing the prominent role it plays in RUE optimization. Thus, providing a new perspective to our understanding of codon usage bias.

RUE measures the average rate with which a ribosome incorporates amino acids into the nascent protein (Eq. 6). Therefore, faster codon translation is necessary for achieving a high RUE (57). A relatively faster codon translation is achieved by codon usage bias and minimization of ribosome traffic-jams (6). Perhaps, this is the reason that highly expressed transcripts have a stronger codon usage bias. Also, the ribosome traffic-jams are minimized at low initiation rates by decreasing the overall ribosome density on a transcript (11). Therefore, the translation–initiation rates are much slower than the average codon translation rate of a transcript. Earlier, low initiation rates were only subjected to the limited availability of ribosomes (58). However, this study shows that it is also a consequence of the optimization of cellular resources.

Highly expressed transcripts tend to have a relatively faster translation initiation (59, 42, 60). However, despite faster initiation, these transcripts have stronger optimization of ribosome utilization (Fig. 3). This observation seems to be inconsistent with our result which shows that high initiation rates decrease the RUE of a transcript (Fig. S7). However, highly expressed transcripts are also enriched with optimal codons which are translated at a faster rate than others (42, 61). This means, ribosomes translating those transcripts can quickly complete the translation process, decreasing the ribosome density, and thus the chances of ribosome traffic jams. Therefore, optimal transcripts with higher codon translation rates can afford to have relatively faster translation initiation rates.

An important implication of this work is in heterologous gene expression which is often used to produce high-quality proteins required for commercial and other purposes (62–64). In this approach, a gene that codes for the desired protein is inserted into a host organism, and then the protein synthesis machinery of the host organism synthesizes that protein (65). However, mere inserting a gene is not sufficient; it has to be optimized as well. Otherwise, little to no protein will be produced (66). A gene for heterologous protein synthesis is often optimized for high TE (67). However, achieving high TE may compromise the translation of other endogenous transcripts by diverting a disproportionate amount of resources for the synthesis of heterologous proteins (64, 65, 11). Therefore, a better strategy is to optimize ribosome utilization than TE. However, the optimization of ribosome utilization may decrease the overall protein production which can be compensated by increasing the copy number of heterologous transcripts.

We find that RUE of the overwhelming majority of transcripts is close to the maximum RUE that a transcript can attain (Fig. 8B). We also find that codon usage bias plays a prominent role in RUE optimization (Fig. 5A). However, in many cases, slow translating codons support different functional requirements of nascent proteins, including co-translational protein folding, signal recognition for signal recognition particle (SRP) binding, and regulation of mRNA half-life (68–72). Therefore, a trade-off is possible between ribosome utilization and the efficiency of other co-translational processes which require further investigations.

We show that non-randomness in translation initiation and codon usage bias optimizes ribosome utilization (Fig. 4). We show this by studying the effects of the elimination of the biases in codon usage and initiation rate on ribosome utilization. We also find that the selection pressure on codon usage bias is much stronger than translation initiation as eliminating codon bias has a more severe impact on RUE as compared to TE (Fig. 5). Moreover, translation initiation rate is mainly determined by nucleotide sequence in the $5^{\prime }$untranslated region (UTR) region (73, 74, 42, 68) whereas codon usage bias is a phenomenon that is limited to only coding sequence. Therefore, these results also highlight co-evolution of UTR and coding sequence of mRNA molecules which requires further investigations.

TE is a parameter that is believed to be optimized for protein synthesis and codon usage bias is thought to play a crucial role in this optimization (75, 67, 76, 8). In fact, a large number of studies have linked codon usage bias with TE (77, 75, 78, 76). However, our study has shown that there is a stronger selection pressure for optimizing RUE than the TE. It is because optimizing ribosome utilization is more effective in reducing the overall energetic cost of protein synthesis than optimizing TE. Note well, we do not claim that TE is not optimized in highly expressed transcripts. However, it is possible that the optimization of TE is just a by-product of the optimization of ribosome usage (Eq. 6). Thus, our study provides a new perspective to the resource optimization in protein synthesis, whose impact on the overall translation landscape requires further investigation.

## Supplementary Material

pgad074_Supplementary_Data

## Data Availability

All data are presented within the manuscript or are available in the Supplementary Material.
