# Supplementary material for: Optimization of ribosome utilization in Saccharomyces cerevisiae
Source: PNAS Nexus. 2023 Mar 9;2(3):pgad074. doi: 10.1093/pnasnexus/pgad074 (PMC10053027; doi:10.1093/pnasnexus/pgad074)
Supplement: pgad074_Supplementary_Data [file pgad074_supplementary_data.pdf]

Supporting information: Optimization of ribosome utilization in *S.cerevisiae*

Mahima[1] and Ajeet K. Sharma[1,2,*]
Department of Physics, Indian Institute of Technology Jammu, 181221
Department of Biosciences and Bioengineering, Indian Institute of Technology Jammu 181221

*corresponding email: ajeet.sharma@iitjammu.ac.in

Supporting results:

There is a systematic difference between RUE and TE. By simulating protein synthesis using the codon translation and initiation rates reported in Ref. (1) and (2), we show that RUE is not a representative of TE and vice versa (main text). Here, we test the robustness of these results by simulating protein synthesis using the initiation and codon translation rates measured in Ref. (3). To this end, we carried out two different tests. First, we test whether the genes with high TE also have a high RUE. In this test, if the TE of a gene is greater than the transcriptome-wide median, then the gene is considered as a high TE gene; otherwise, the gene has a low TE. Similarly, we label all genes with high or low RUE genes. To clearly visualize this, we divide Fig. S12 in four different regions by the lines separating them at median RUE and TE. We find that 50.35% of the total genes are either in the left top or bottom right regions of Fig. S12. In those genes, RUE (or TE) is high but TE (or RUE) is low. This means that a low or high TE does not ensure a low or high RUE, respectively. We also compared the TE and RUE of each transcript with the rest of the other transcripts. We find that, 417408 out of the 827541 gene pairs (*i.e.*, over 50.44% of the total gene pairs), a gene with a larger (or smaller) TE has a smaller (or larger) RUE.

Second, if RUE is a representative of TE, then both of these parameters should follow the same distribution because there should be no systematic differences between them. To test this, we first normalize TE and RUE with respective mean values and compute their probability distribution (Fig. S13). Then, we test whether the normalized TE and RUE follow the same distribution by applying the Kolmogorov Smirnov test. The test rejects the null hypothesis that normalized TE and RUE are distributed in a similar manner (p-value = 6.3785e-80). These results show that there is a systematic difference between RUE and TE, and they cannot be used interchangeably.

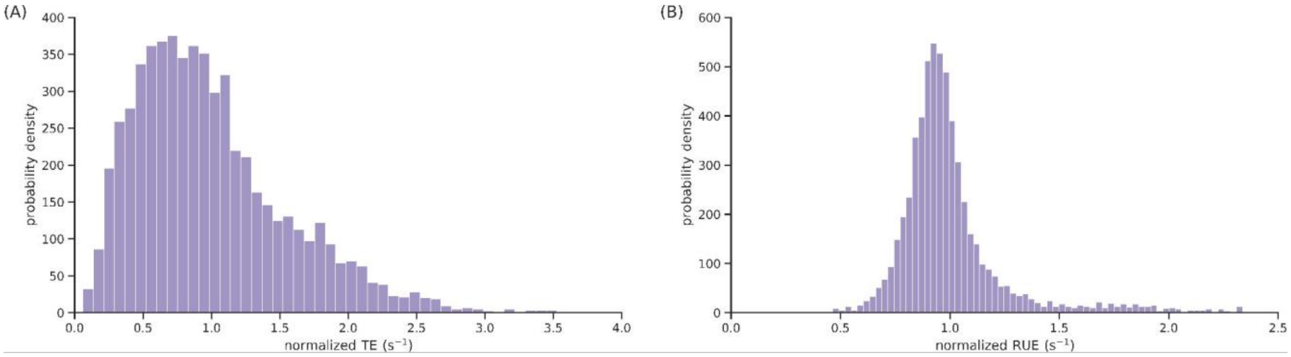

Fig. S1: The probability distribution of normalized TE and RUE are plotted in (A) and (B), respectively. TE and RUE are computed by simulating protein synthesis using the translation-initiation and codon translation rates reported in Refs. (1) and (2), respectively.

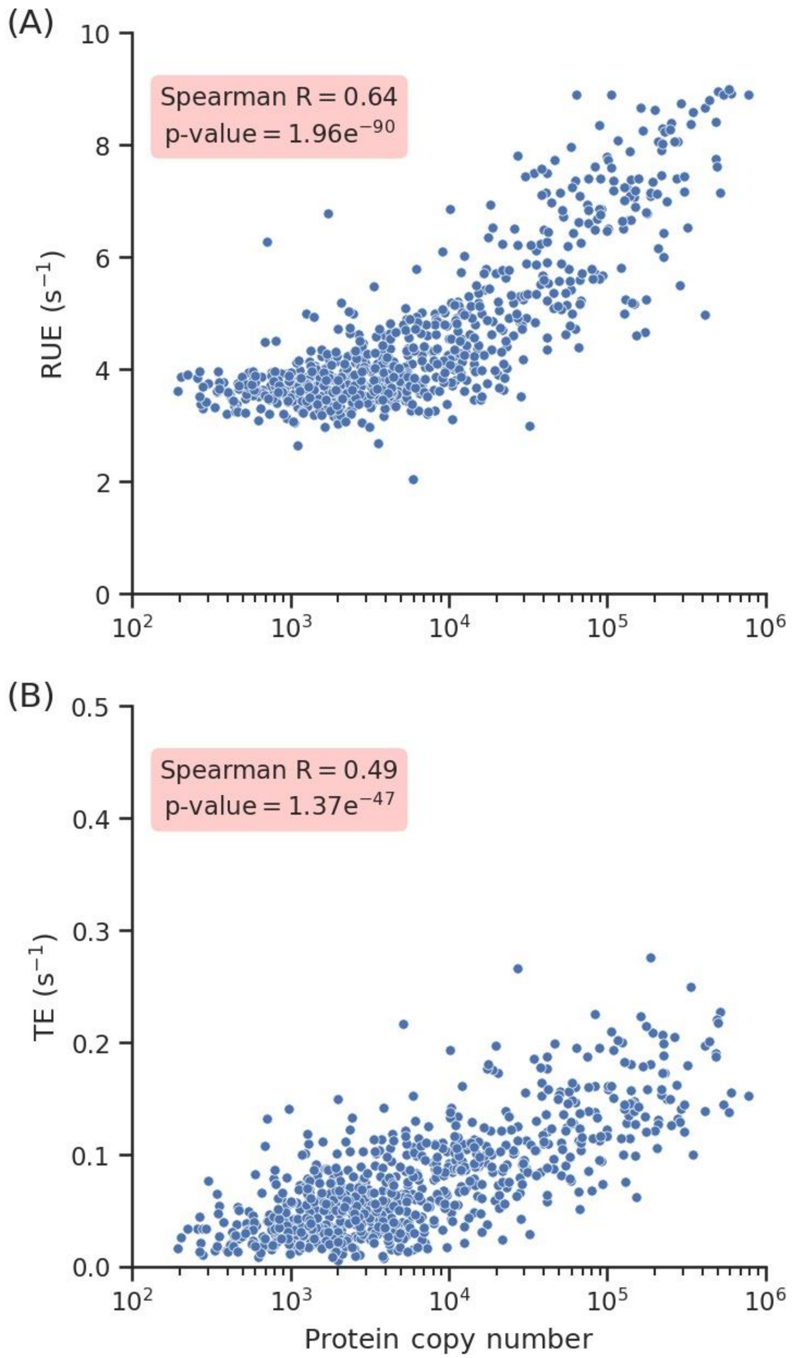

Fig. S2: Scatter plots of RUE and TE with protein copy number in (A) and (B), respectively. RUE and TE in this figure are computed by simulating protein synthesis using the translation-initiation and codon translation rates reported in Refs. (1) and (2), respectively. The protein copy numbers are from the Ref. (4).

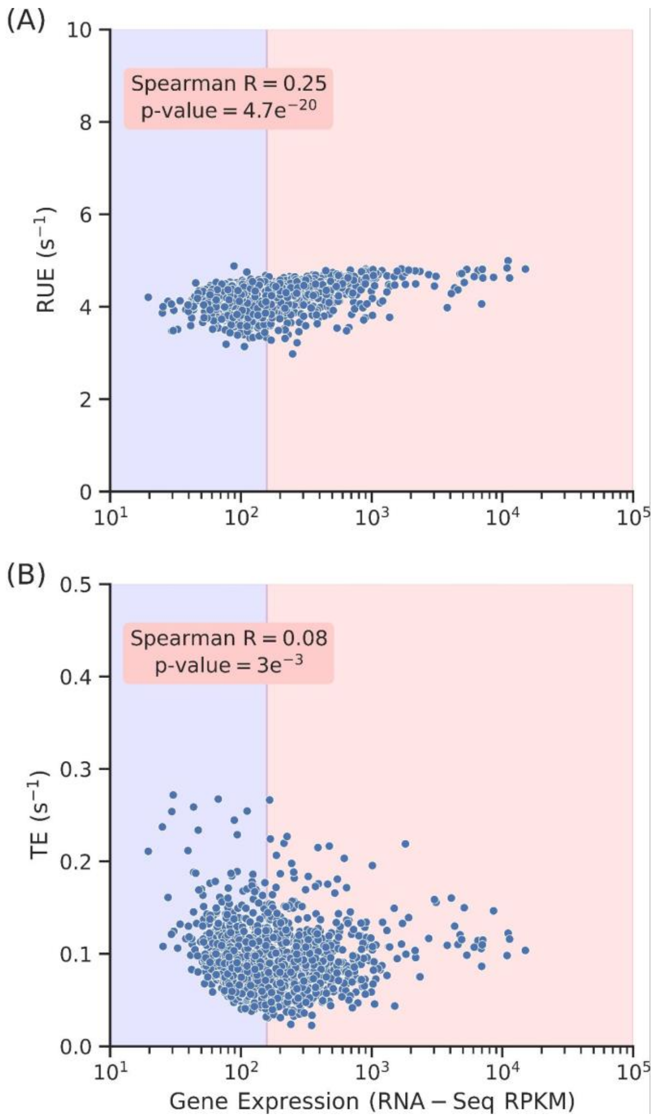

Fig. S3 : RUE is better optimized than TE. RUE and TE are plotted against mRNA abundance in (A) and (B), respectively. RUE and TE in this figure are computed by simulating protein synthesis using the translation-initiation and codon translation rates reported in Ref. (3).

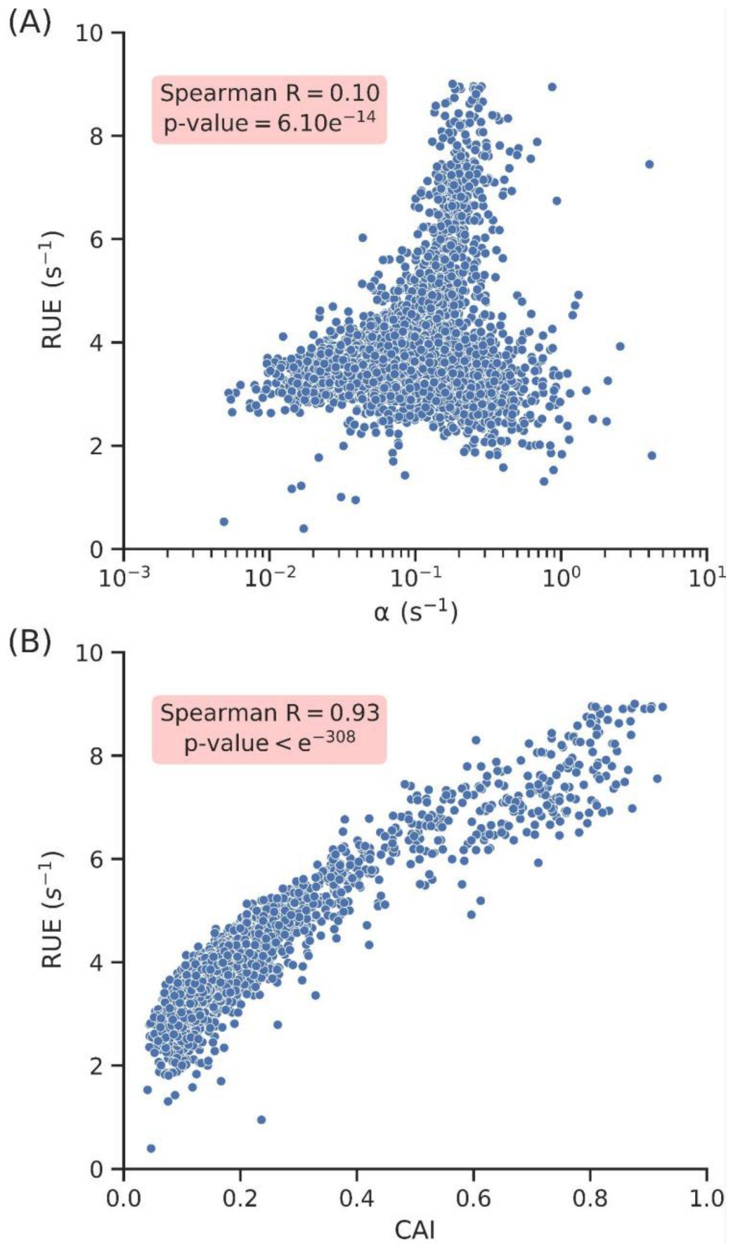

Fig. S4: RUE is plotted against initiation rate and CAI in (A) and (B), respectively. RUE is computed by simulating protein synthesis using translation-initiation and codon translation rates as reported in Refs. (1) and (2). CAI values are from Ref. (4).

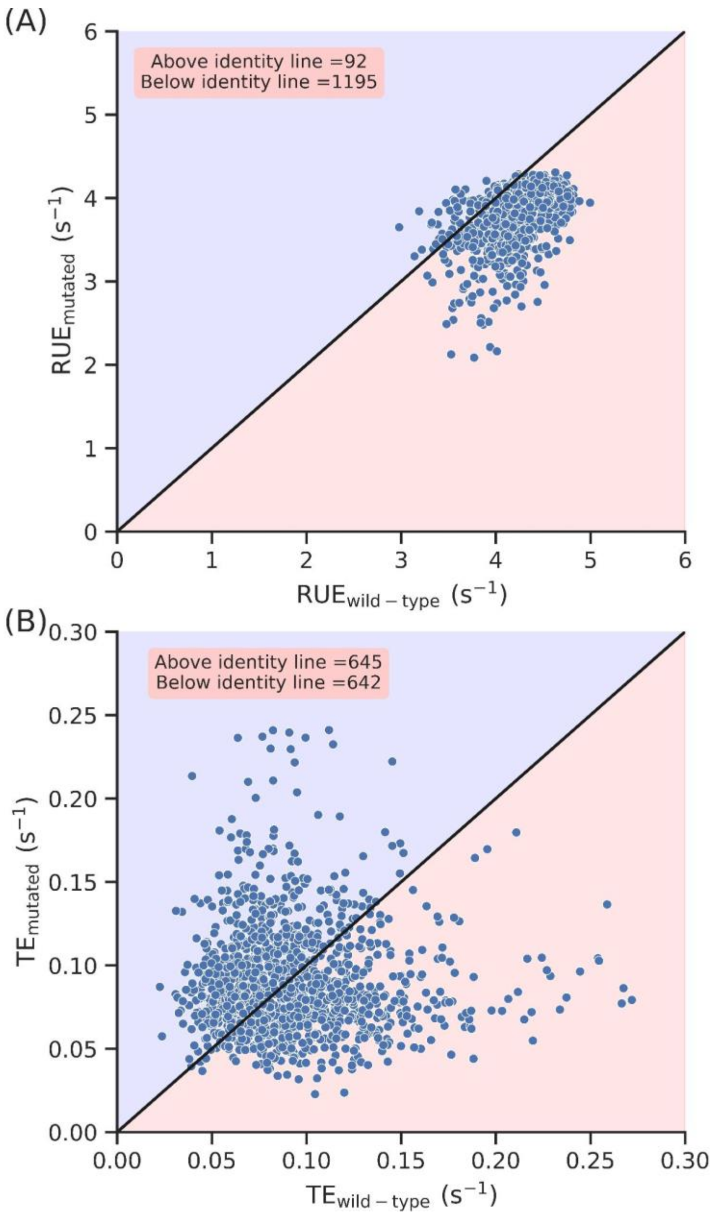

Fig. S5: RUE and TE of mutated transcripts are plotted against RUE and TE of the wild-type transcripts in (A) and (B), respectively. RUE and TE in this figure are computed by simulating protein synthesis using the translation-initiation and codon translation rates as reported in Ref. (3).

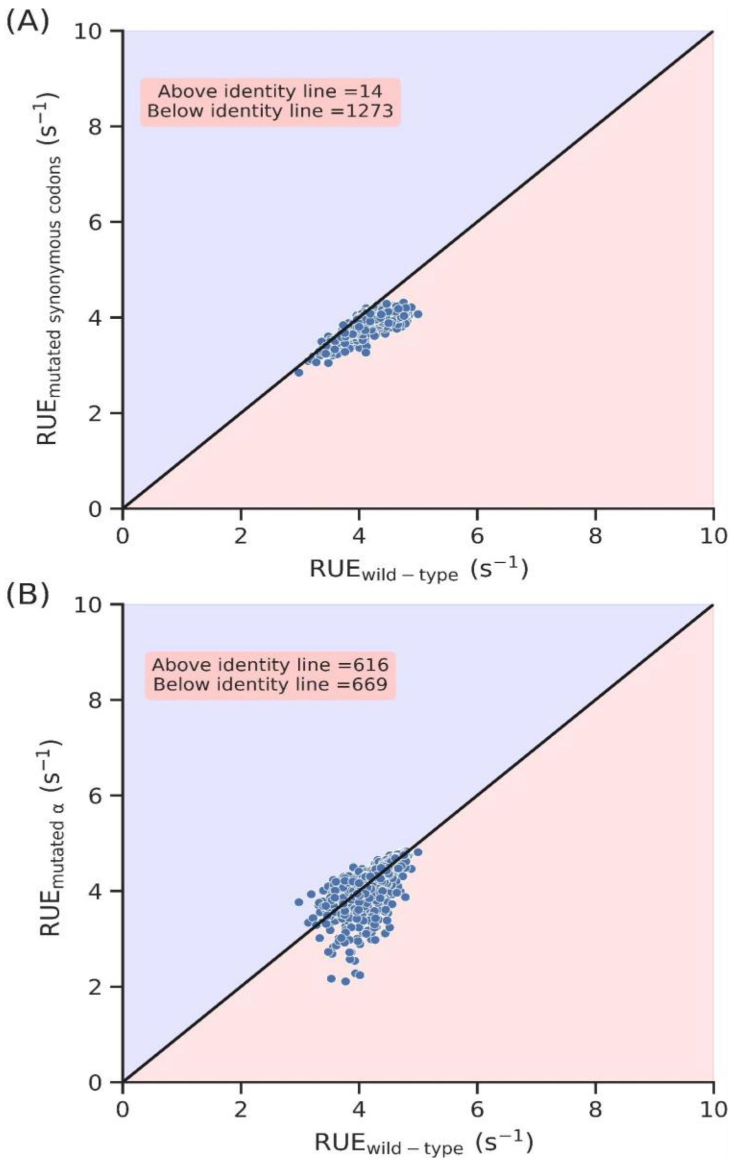

Fig. S6: Selection pressure on optimizing codon usage bias is much stronger than translation initiation rate. (A) RUE of the wild-type transcripts are plotted against the RUE of the same transcript when all of its codons are mutated with randomly selected synonymous codons.(B) RUE of the wild-type transcripts are plotted against the RUE of the same transcript with randomly assigned initiation rate. We computed RUE and TE by simulating protein synthesis using the translation-initiation and codon translation rate reported in Refs. (1) and (2).

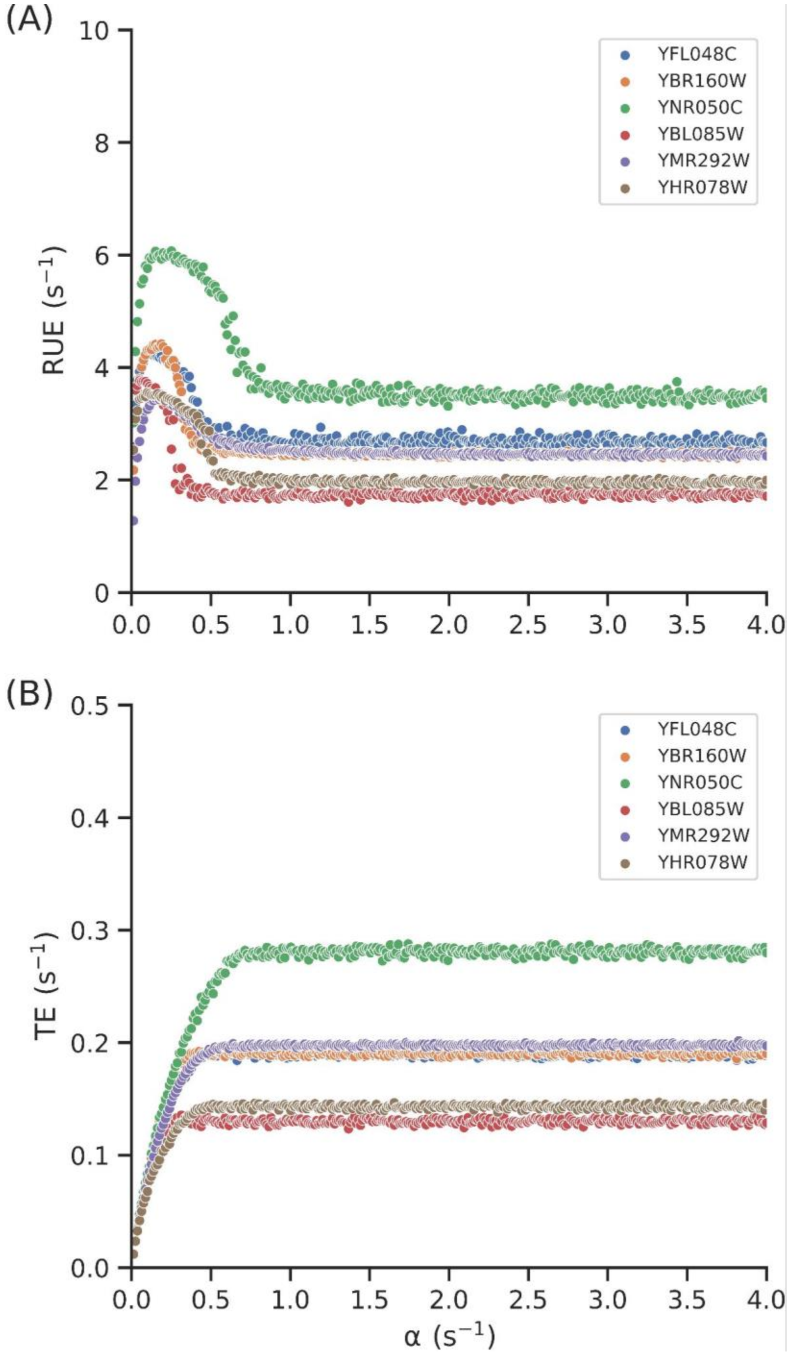

Fig. S7: TE and RUE for six randomly selected transcripts are plotted as a function of initiation rate in (A) and (B), respectively. (A) As protein synthesis transitions from the LD to MC regime, the TE increases and then saturates. (B) Whereas slight humps, showing a maximum value of RUE, are observed as α varies from 0.00 to 4.00 s^{-1}.

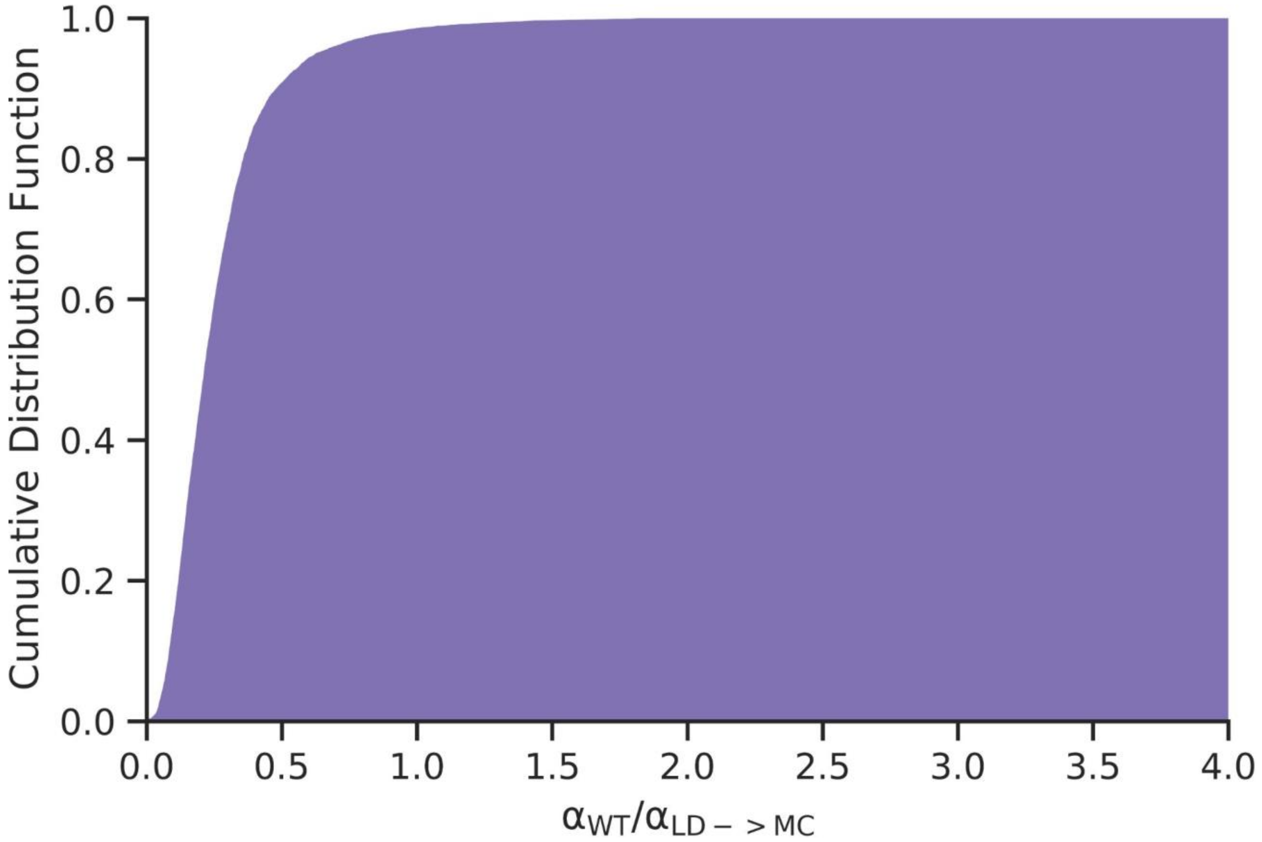

Fig. S8: Cumulative distribution of the ratio of wild-type initiation rate is plotted against the initiation rate that maximizes TE. The ratio is less than 0.75 for more than 95% of genes. Thus, indicating that wild-type initiation rates are much lower than initiation rate where regime changes from LD to MC. We have used wild-type translation-initiation rates as reported in Ref. (1).

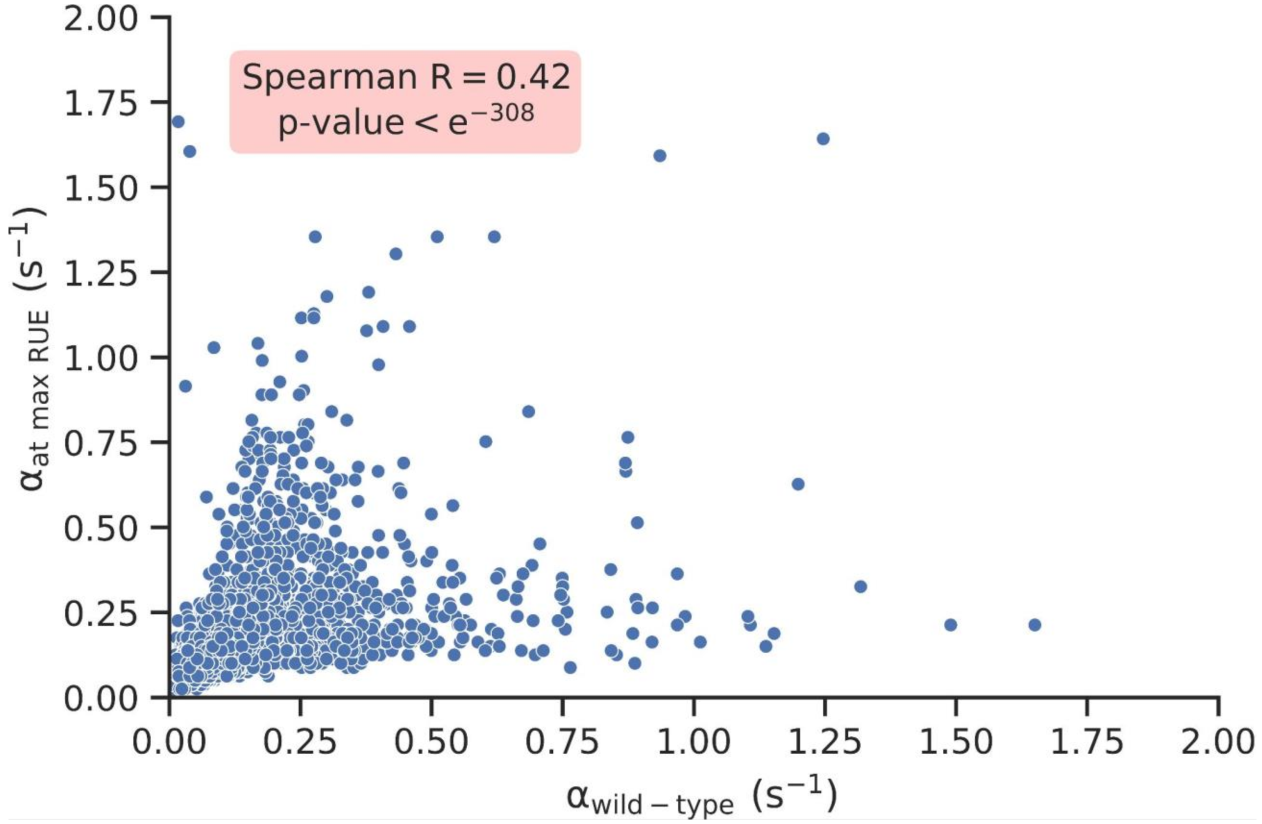

Fig. S9: The wild-type initiation rates are plotted against the initiation rates that maximize RUE. In this figure, we used the wild-type translation-initiation rates as reported in Ref. (1).

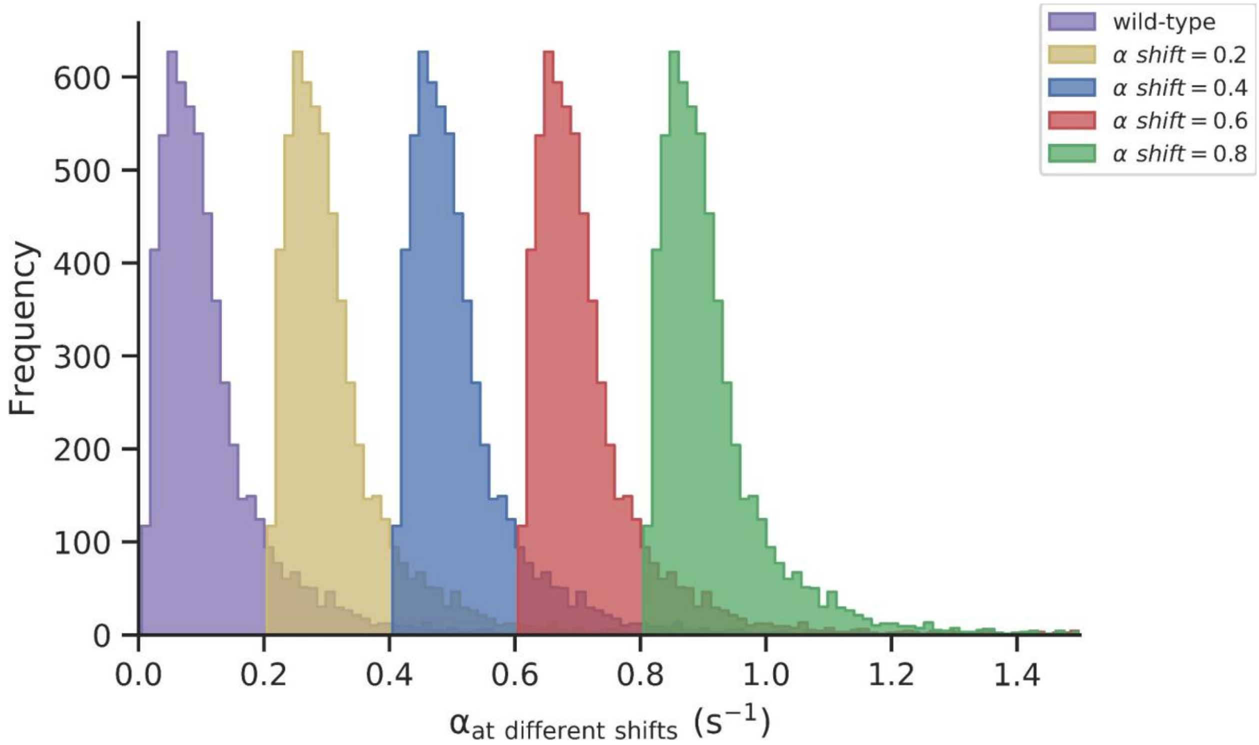

Fig. S10: The distribution of wild-type initiation rate (Ref. (1)) and four other sets of transcripts are plotted. These four other sets are created by shifting the wild-type initiation rates by 0.2, 0.4, 0.6 and 0.8 s^{-1}.

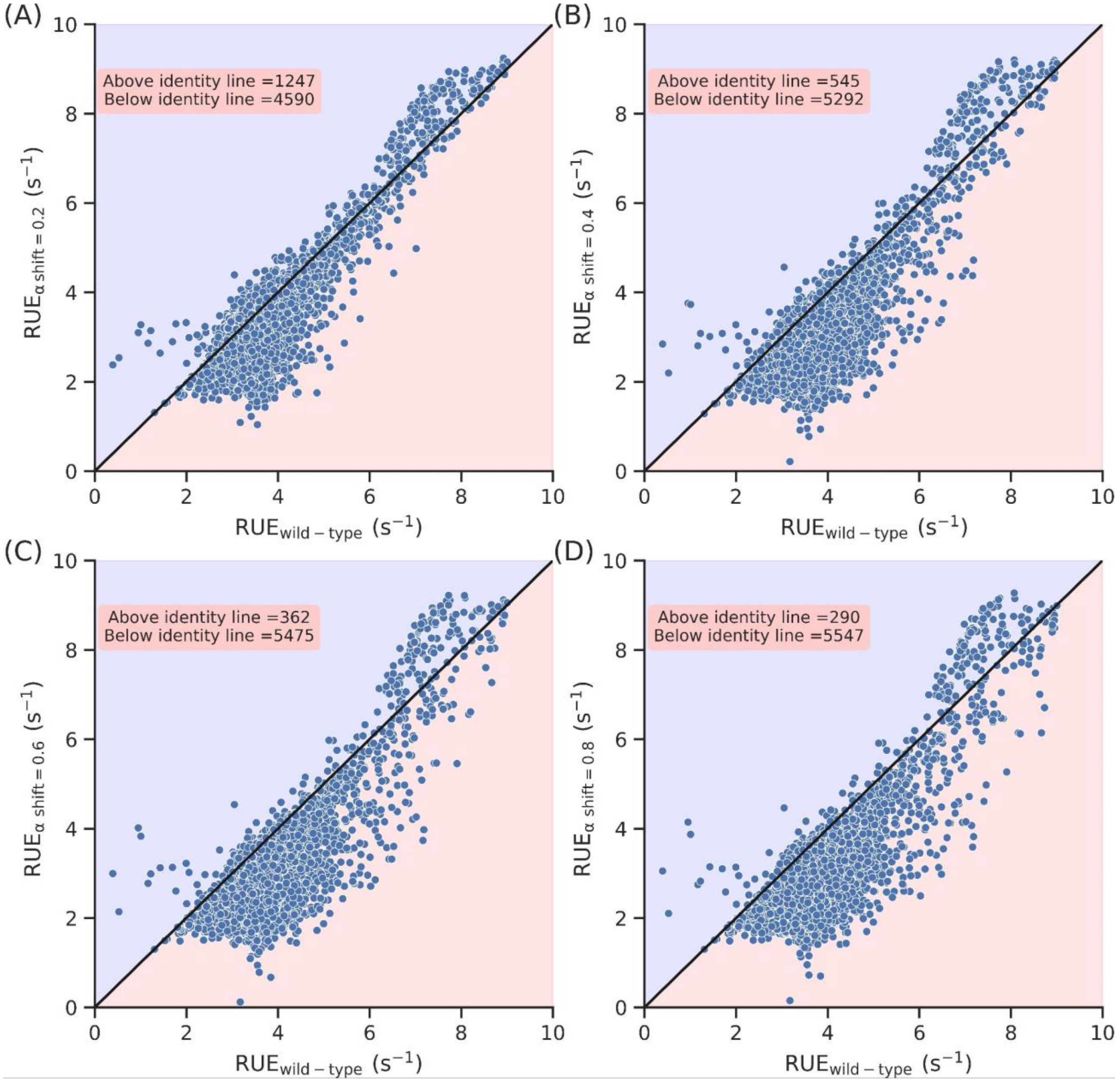

Fig. S11: Wild-type RUE is plotted against the RUE of the same transcript when their initiation rates are shifted by 0.2, 0.4, 0.6 and 0.8 s^{-1} in (A), (B), (C) and (D), respectively.

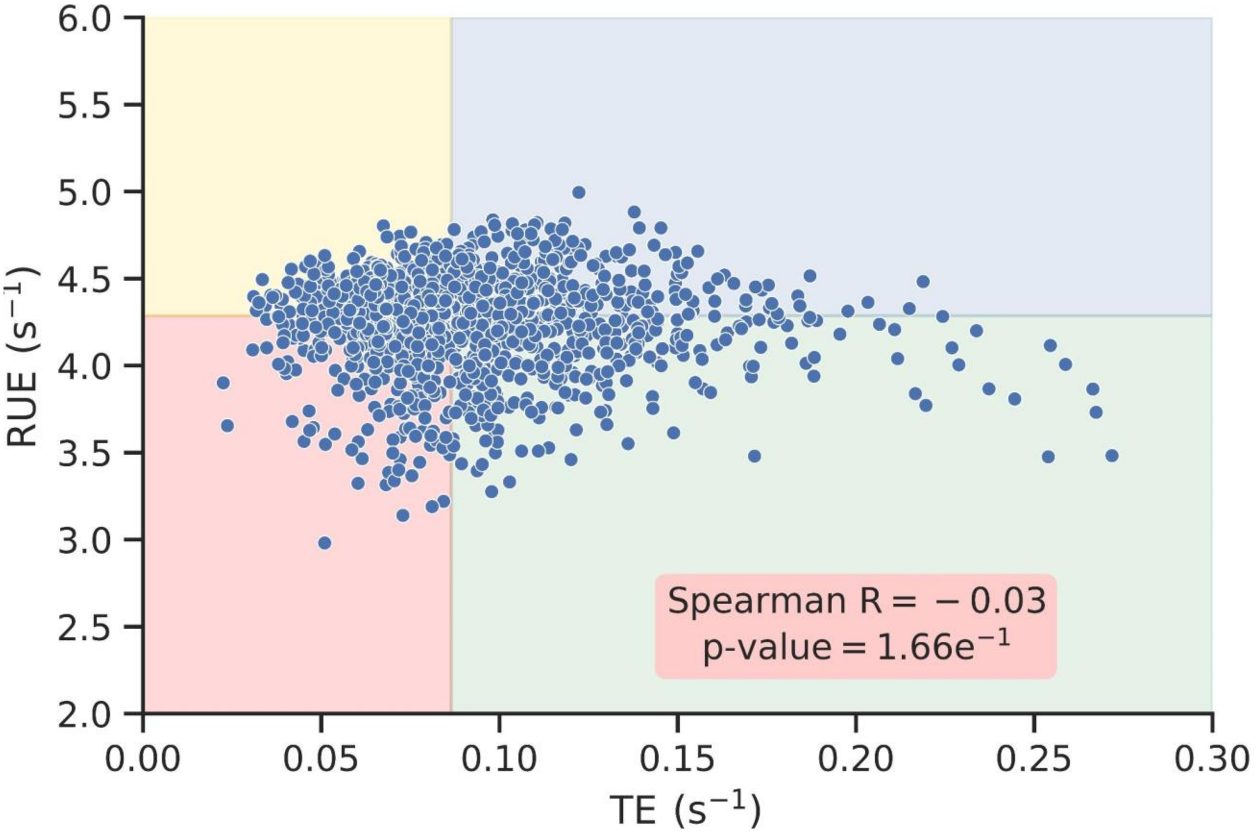

Fig. S12: RUE of *S. cerevisiae* transcripts are plotted against TE. The figure consists of four subsections which are created by the lines separating them at median TE and RUE. For each transcript, RUE and TE are computed by simulating protein synthesis using the translation-initiation and codon translation rate as reported in Ref. (3).

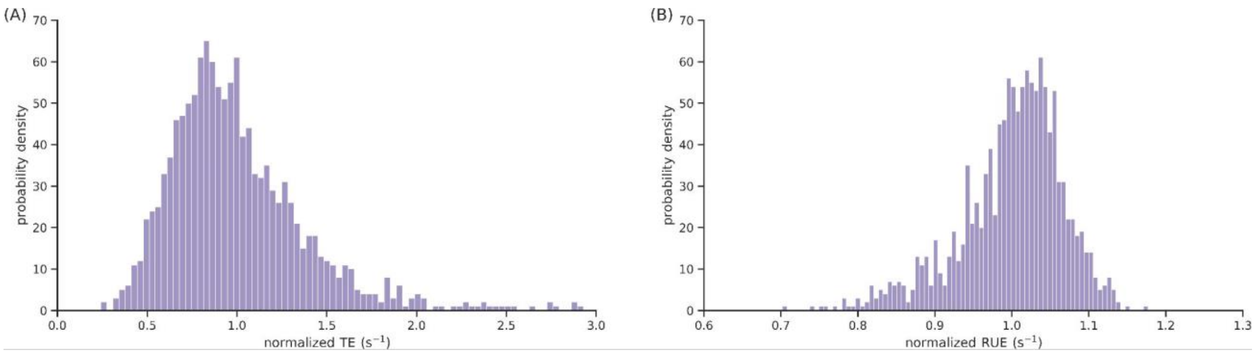

Fig. S13: The probability distribution of normalized TE and RUE are plotted in (A) and (B), respectively. TE and RUE are computed by simulating protein synthesis using the translation-initiation and codon translation rates as reported in Ref. (3).

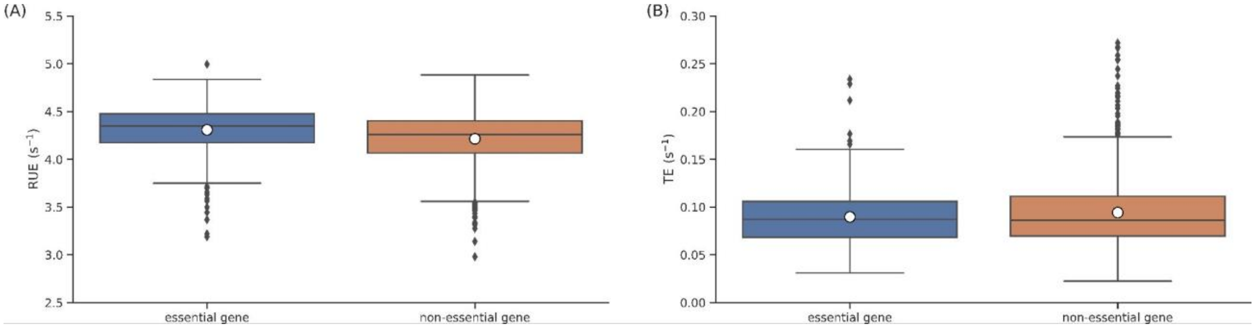

Fig. S14: Box-plots illustrating RUE and TE of essential and non-essential genes are shown in (A) and (B), respectively. The average RUE in essential genes is 2.2% higher than the other transcripts (Wilcoxon rank-sum test, p-value = 2.5092e-09), whereas no statistically significant difference was observed between TE of the essential and non-essential genes. (Wilcoxon rank-sum test, p-value = 0.2920). In this figure, we use the list of essential and non-essential genes reported as in Ref. (5).

	No. of genes for which $RUE_{wild\text{-}type}$ > $RUE_{mutated}$	No. of genes for which $RUE_{wild\text{-}type}$ < $RUE_{mutated}$	No. of genes for which $TE_{wild\text{-}type}$ > $TE_{mutated}$	No. of genes for which $TE_{wild\text{-}type}$ < $TE_{mutated}$	% decrement in Ribosome requirement by introducing non-randomness in initiation rates and eliminating codon usage bias
Dataset 1	5216	621	3012	2824	53.89%
Dataset 2	5186	651	3025	2812	51.98%
Dataset 3	5229	608	3024	2812	54.61%
Dataset 4	5182	655	3046	2790	53.89%
Dataset 5	5207	630	3047	2790	52.07%
Dataset 6	5206	631	2999	2838	53.78%
Dataset 7	5184	653	3032	2805	52.02%
Dataset 8	5200	637	3004	2833	53.03%
Dataset 9	5212	625	3033	2803	52.23%
Dataset 10	5182	655	3055	2782	53.10%
Dataset 11	5198	639	3035	2802	52.15%
Dataset 12	5170	667	3039	2798	52.77%
Dataset 13	5192	645	3053	2783	52.49%
Dataset 14	5170	667	3030	2807	54.22%
Dataset 15	5209	628	3043	2794	53.95%
Dataset 16	5194	643	3057	2780	53.78%
Dataset 17	5195	642	3003	2834	55.07%
Dataset 18	5190	647	3026	2811	53.77%
Dataset 19	5202	635	3013	2823	51.56%
Dataset 20	5201	636	3018	2819	52.08%

Table S1: Quantitative analysis listing the number of genes for which the wild-type RUE and TE is greater or less than the RUE and TE of mutated ones. The data is obtained by plotting RUE and TE of mutated transcripts against wild-type RUE and TE for all datasets.

References

(1). L Ciandrini, I Stansfield, MC Romano, Ribosome traffic on mrnas maps to gene ontology: genome-wide quantification of translation initiation rates and polysome size regulation. PLoS computational biology 9, e1002866 (2013).
(2). A Fluitt, E Pienaar, H Viljoen, Ribosome kinetics and aa-trna competition determine rate and fidelity of peptide synthesis. Comput. biology chemistry 31, 335–346 (2007).
(3). AK Sharma, et al., A chemical kinetic basis for measuring translation initiation and elongation rates from ribosome profiling data. PLoS computational biology 15, e1007070 (2019).
(4). P Lu, C Vogel, R Wang, X Yao, EM Marcotte, Absolute protein expression profiling estimates the relative contributions of transcriptional and translational regulation. Nat. biotechnology 25, 117–124 (2007).
(5). G Liu, et al., Gene essentiality is a quantitative property linked to cellular evolvability. Cell 163, 1388–1399 (2015).
